# Integrated analysis of tobacco miRNA and mRNA expression profiles under PVY infection provids insight into tobacco-PVY interactions

**DOI:** 10.1038/s41598-017-05155-w

**Published:** 2017-07-07

**Authors:** Yushuang Guo, Meng-ao Jia, Yumei Yang, Linlin Zhan, Xiaofei Cheng, Jianyu Cai, Jie Zhang, Jie Yang, Tao Liu, Qiang Fu, Jiehong Zhao, Imran Haider Shamsi

**Affiliations:** 1 Key Laboratory of Molecular Genetics, China National Tobacco Corporation, Guizhou Institute of Tobacco Science, Guiyang, Guizhou 550083 P. R. China; 2 Annoroad Gene Technology (Beijing) Co., Ltd, Beijing, 101100 P. R. China; 3 Department of Agronomy, College of Agriculture and Biotechnology, Zhejiang University, Hangzhou, Zhejiang 310058 P. R. China; 4 College of Agriculture and Food Science, Zhejiang Agriculture and Forestry University, Hangzhou, Zhejiang 311300 P. R. China; 5 School of Life and Environmental science, Hangzhou Normal University, Hangzhou, Zhejiang 311121 P. R. China

## Abstract

*Potato virus Y* (PVY) is a globally and economically important pathogen of potato, tobacco, tomato and other staple crops and caused significant yield losses and reductions in quality.To explore the molecular PVY-host interactions, we analysed changes in the miRNA and mRNA profiles of tobacco in response to PVY infection. A total of 81 differentially expressed miRNAs belonging to 29 families and 8133 mRNAs were identified. The Gene Ontology (GO) enrichment analyses showed that genes encoding the DNA/RNA binding, catalytic activity and signalling molecules were all significantly enriched. Moreover, 88 miRNA-mRNA interaction pairs were identified through a combined analysis of the two datasets. We also found evidence showing that the virus-derived siRNAs (vsiRNAs) from the PVY genome target tobacco translationally controlled tumor protein (*NtTCTP*) mRNA and mediate plant resistance to PVY. Together, our findings revealed that both miRNA and mRNA expression patterns can be changed in response to PVY infection and novel vsiRNA-plant interactions that may regulate plant resistance to PVY. Both provide fresh insights into the virus-plant interactions.

## Introduction

*Potato virus Y* (PVY) is an economically important pathogen of many crops, with many of its strains differing from one another both in their genomic sequences and in the symptoms they produce in their hosts^1–3^. The PVY genome is a positive-sense, single-stranded RNA molecule consisting of approximately 9700 nucleotides. The PVY genome contains two open reading frames (ORFs). The first ORF is translated into a single polyprotein and then processed into individual mature proteins by the viral proteases. The second—and shorter—ORF is translated as the P3N–PIPO fusion protein which is embedded within the P3 cistron of the polyprotein^4^.

Small RNAs are a group of regulatory molecules that play important roles in diverse biological processes, namely in development, genome maintenance and integrity, and in the adaptive responses to biotic and abiotic stress in most of the eukaryotes. Small RNAs are of two major types: microRNAs (miRNAs) and short interfering RNAs (siRNAs), both function by suppressing the expression of target genes at the transcriptional and/or post-transcriptional level via specific base-pairing with their targets^5^. As a key compontent of the eukaryotic gene regulatory networks, miRNA has attracted increasing attention with respect to its biogenesis and mechanisms of miRNA-mediated gene regulation^6–8^. It was reported that some animal cellular miRNAs play important roles in the proess of development and the response to pathogens and stresses^6, 7^. Many animal viruses can down-regulate the expression level of host miRNAs^9–13^. Deep sequencing has also identified a few new miRNAs induced only in virus infected-cells^14, 15^. In *Arabidopsis*, the activation of antiviral RNAi is accompanied by the production of an abundant class of endogenous siRNAs mapped to the exon regions of more than 1,000 host genes and rRNA. These virus-activated siRNAs are predominantly 21 nucleotides in length, with an approximately equal ratio of sense and antisense strands, and they may confer broad-spectrum antiviral activity^16^.

As a part of small RNA, virus-derived small interfering RNA (vsiRNA), is abundant during the viral infection in plants. Although double-strand replication intermediates (RIs) could form in the process of virus multiplication, the dsRNA-like secondary structures of single stranded viral RNAs were those that most likely contributed to vsiRNA biogenesis^17–20^. Analogous to that of endogenous small RNA, the biogenesis of vsiRNA requires not only Dicer-like (DCL) —especially DCL4 which processes the viral dsRNA transcript into primary vsiRNA^21, 22^—but also (RNA- dependent RNA polymerase (RDR) and Argonaute (AGO) which produced secondary vsiRNA through amplification^23–25^. Biogenesis of vsiRNA during virus infection indicates that vsiRNA may function in many regulation pathways. For instance, vsiRNA could be recruited by diverse AGOs to form RNA-induced silencing complex (RISC) and target viral genome molecules (including viral RNA and viral DNA) through post-transcriptional gene silencing (PTGS)^21, 26^, and vsiRNA might also target and down-regulate host transcripts that largely determine the virus symptoms in the host^27–30^.

To date, the responses of plants to PVY infection have until now been studied at different levels, ranging from morphological to biochemical, and from proteomic to transcriptomic and metabolic^31–35^. Nevertheless, our knowledge of how plants respond to PVY infection remains rather limited. Hence, further investigation is warranted to fully explore the plant-virus interaction dynamics behind the appearance of disease symptoms and the plant resistance processes.

To acquire a better understanding of how the transcriptome changes in response to viral infection in tobacco, we used high-throughput sequencing technology to simultaneously analyse the miRNA and mRNA expression profiles in virus-infected tobacco plants. We combined these two datasets and identified miRNA-mRNA interactions under PVY infection. We also found that the vsiRNAs from PVY target tobacco *NtTCTP* mRNA and mideate plant resistance to PVY infection.These integrated high-throughput expression data provide a new and valuable resource for examining global genome expression changes in response to PVY infection. This may contribute to viral symptom development and thereby provide new insights into plant-virus interactions.

## Results

### Construction and deep sequencing of small RNA and mRNA libraries

To profile the global small RNA and mRNA changes via deep sequencing, the total RNAs were isolated from the PVY-inoculated (three biological replicates: A1, A2, A3) and mock-inoculated (three biological replicates: B1, B2, B3) tobacco plants, and used to construct small RNA and transcriptome libraries. All the data generated by this deep sequencing exercise were uploaded to the SRA database (Accession number: SRP090053).

**Figure 1:**
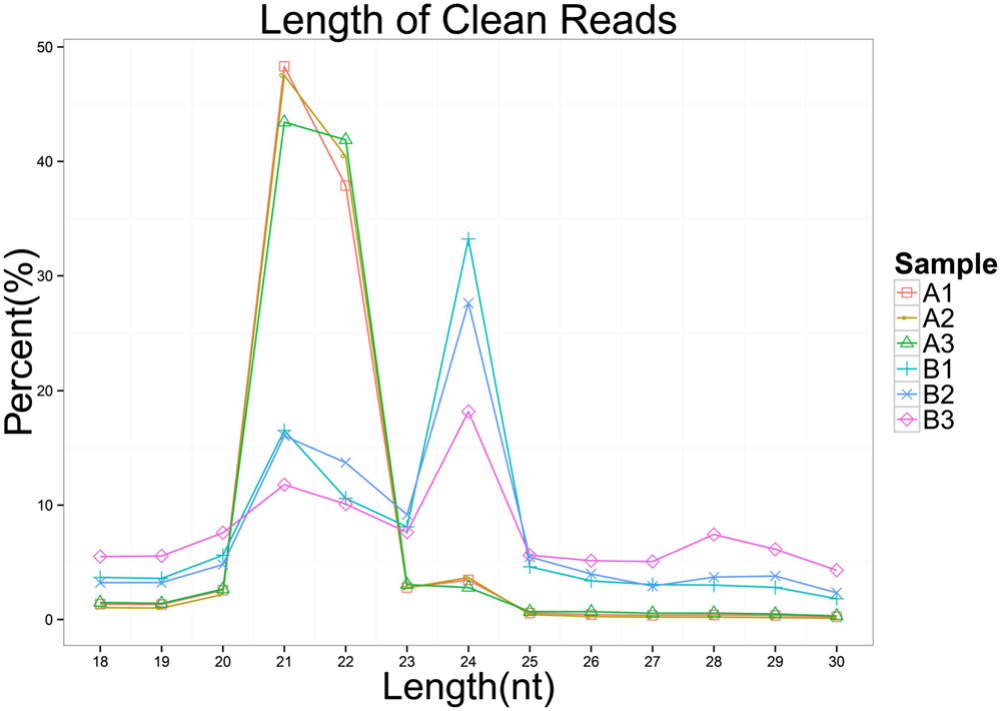
Length distribution of tags in the small RNA libraries. The relative abundance of small RNA with length ranging from 18–30 nt are shown in the graph.

Small RNA sequencing generated approximately 1 million raw reads from each library. After the 5′ and 3′ adaptors were identified and removed from the raw reads, those reads with a sequence length of 18 to 30 nt were selected for further analysis (Table 1). The distribution of small RNAs among the different categories is summarized in Fig.1. In the PVY-infected plants, 21 nt and 22 nt are the most frequent sizes. By contrast, in the mock-inoculated plants, the most frequent small RNAs were 21 nt and 24 nt in length. More than 75% of the small RNA sequences that had a perfect match to the tobacco genome sequence (https://solgenomics.net) were obtained from each library. In the small RNA libraries of the PVY-infected tobacco plants, a similar percentage of high-quality read and unigene were found well matched to the PVY genome.

**Table 1:** The Small RNA sequencing results of PVY-infected and mock-inoculated tobacco.

Libraries	Replicate1		Replicate2		Replicate3	
	PVY(A1)	Mock(B1)	PVY(A2)	Mock(B2)	PVY(A3)	Mock(B3)
Q20	99.35%	99.12%	99.27%	98.93%	99.27%	98.67%
Q30	97.34%	96.51%	97.17%	96.03%	97.11%	95.05%
GC content	49.55%	50.94%	49.17%	51.24%	49.69%	53.63%
RawReads	22144705	20273746	18966413	19158944	20583285	18294253
RawBases	1.11E + 09	1.01E + 09	9.48E + 08	9.58E + 08	1.03E + 09	9.15E + 08
Total clean reads	21054651	19203943	17983010	17263866	19620809	16084323
Unique clean reads	3172054	6283820	2791966	5986140	3042445	4110929

**Figure 2:**
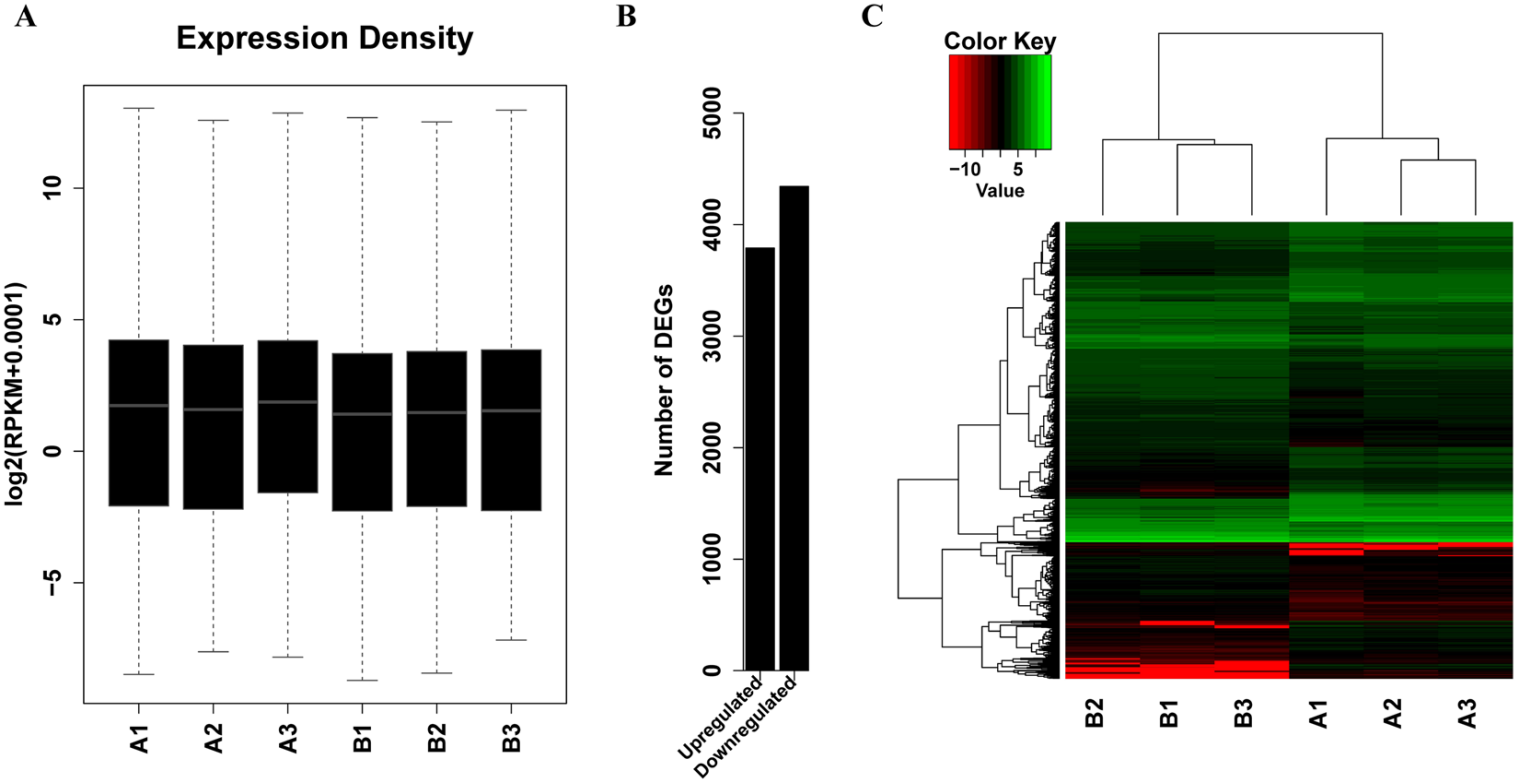
Differentially expressed mRNAs in PVY-infected and mock-inoculated tobacco plants. (**A**) Gene density of mRNAs in the PVY-infected (three biological replicates: A1, A2, A3) and mock-inoculated (three biological replicates: B1, B2, B3) tobacco RNA libraries; (**B**) Total numbers of up-regulated and down-regulated DEGs; (**C**) Differentially expressed mRNAs in PVY-infected (A1, A2, A3) and mock-inoculated (B1, B2, B3) tobacco. Every row shows a different gene. Red, black and green indicates expression levels of mRNA slow, medium and high, respectively.

Approximately 7G of clean reads were obtained from each mRNA library. The mapping rate of all three PVY-infected libraries (A1, A2, A3) was 77–78%, while it was slightly higher for the mock-inoculated libraries (B1, B2, B3) at 84–89%. Meanwhile, the multi-map rate of the samples was less than 0.1% (Table 2). The gene expression density of all the samples showed a high similarity: there were few differential genes, and these usually would not change the overall gene distribution (Fig. 2A). Both the PVY-infected samples and mock-inoculated samples showed good correlations based on the Hierarchical cluster analysis (Fig. 2C).

**Table 2:** Categorization and abundance of tags of PVY-infected and mock-inoculated tobacco RNA libraries.

#Sample	A1	A2	A3	B1	B2	B3
total_read	134659228	84038828	98128294	102376902	120800002	72364734
mapped_reads	103234569	65505578	75602181	91297504	101606947	61068947
mapping_rate	76.66%	77.95%	77.04%	89.18%	84.11%	84.39%
unmapped_reads	31424659	18533250	22526113	11079398	19193055	11295787
multi_map_reads	5716092	4005916	3795713	6469833	7192648	4394154
multi_map_rate	4.24%	4.77%	3.87%	6.32%	5.95%	6.07%

All the data showed good agreement among the replicates. Therefore, the data derived to form high quality small RNA and mRNA libraries were deemed robust for further analysis.

### PVY infection selectively altered the expression of tobacco miRNAs

To speculate on how PVY affects the expression of miRNAs, we compared the total reads of the tobacco miRNAs among the six libraries using pooled data from the three independent biological replicates. The analysis of the miRNA data showed that 322 miRNAs were detected in the PVY-infected and mock-inoculated tobacco plants (Supplementary Table S1), However, only 81 miRNAs changed their expression level significantly in the PVY-infected tobacco, of which 24 miRNAs were down-regulated and 57 miRNAs were up-regulated (Supplementary Fig. S1A, Supplementary Table S2). This suggested that some miRNA, or the locus encoding the miRNA precursor, could respond to the PVY infection. To verify the accuracy of the miRNA alterations by data calculation, several characteristic miRNAs, such as mi156g and mi168a, were selected for confirmation by the northern blot method. These results showed that the differences in the heat map were consistent with those of the northern blot (Supplementary Fig. S1B,C).

**Figure 3:**
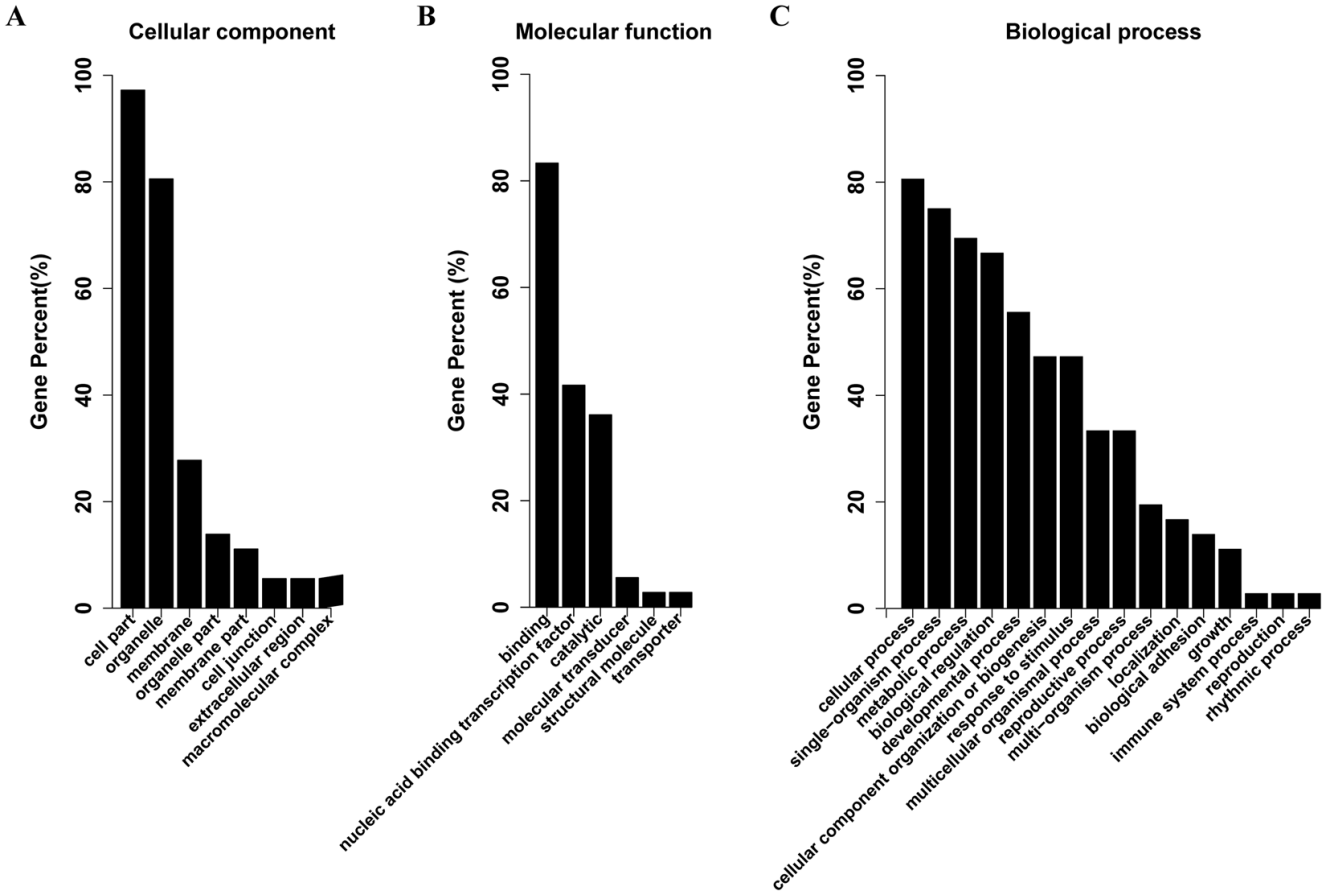
Gene ontology (GO) of predicted targets by differentially expressed miRNAs under PVY infection. (**A**) Category of cellular components; (**B**) Category of molecular functions;(**C**) Category of biological process.

To obtain those genes that possibly regulated by the differentially expressed miRNAs, targets of the miRNAs were predicted by using psRobot software (Supplementary Table S3). To confirm the predicted results, we sequenced a degradome library constructed using the total RNA isolated from the leaf of PVY-infeted tobacco plants. Over 9.9 million of raw reads were thus generated (Accession number: SAMN06844094). The degradome sequencing datasupported the predicted results for the miRNA targets (Supplementary Table S4). GO enrichment analysis of the target genes of significantly differentially-expressed miRNAs (*P*_*adj*_ < 0.05 and |log_2_(fold_change)| ≥ 1) among the paired group samples was performed. The ensuing GO categorization of the predicted targets of the differentially expressed miRNAs showed that these genes were involved in a broad range of biological processes related to cellular responses to various stimuli; namely, the positive regulation of cellular processes, the negative regulation of growth, the nucleic acid binding and the transcription factors requied for signal transduction (Fig. 3A,B,C).

### Global mRNA expression profiles of tobacco in response to PVY infection

**Figure 4:**
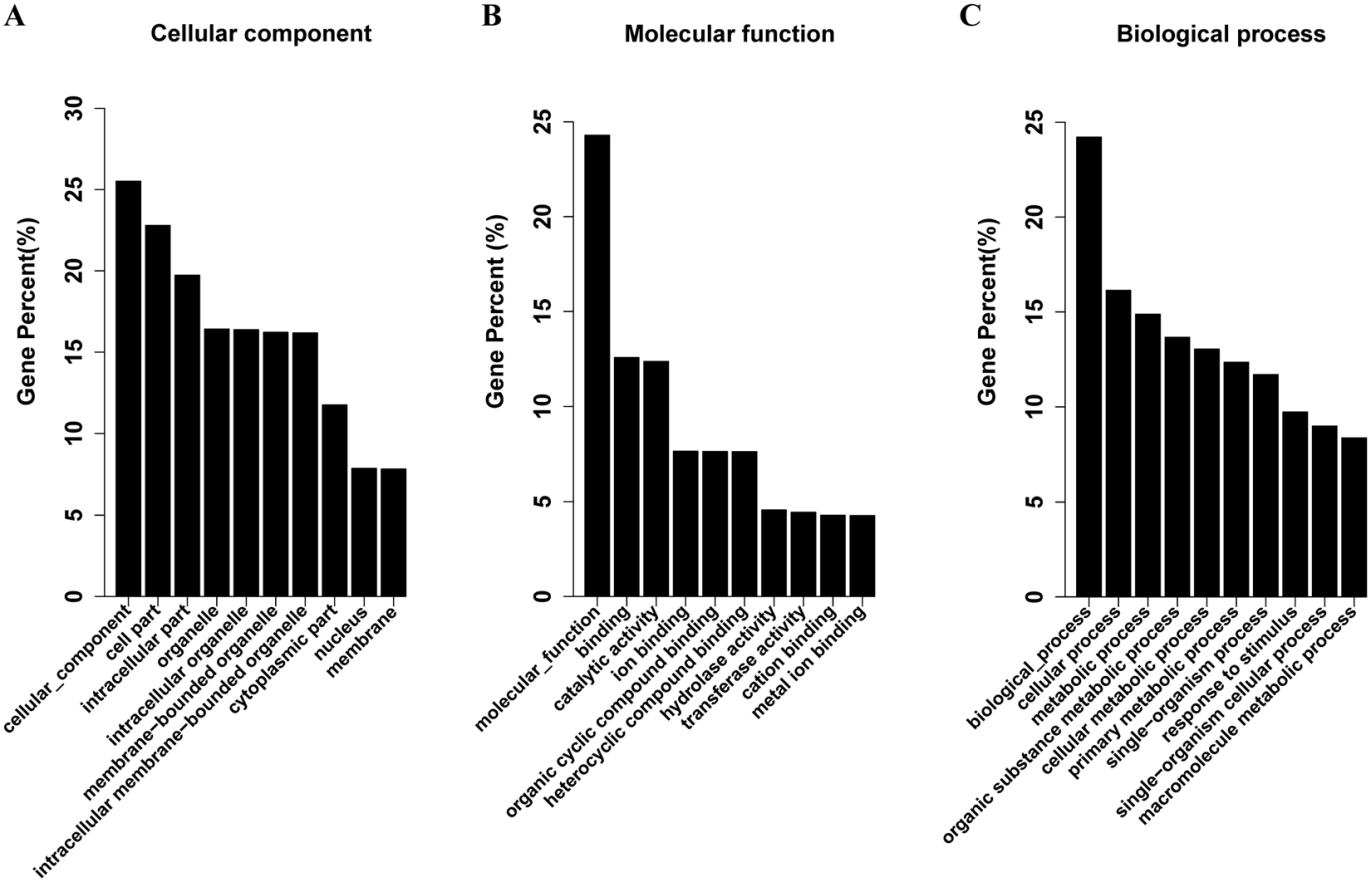
Gene ontology (GO) analysis of differentially expressed mRNAs under PVY infection in tobacco. (**A**) Category of cellular components; (**B**) Category of molecular functions; (**C**) Category of biological processes.

To further study the tobacco gene expression profiles responsive to PVY infection, the total RNA samples used for small RNA sequencing were subjected to transcriptome sequencing. Analysing this deep sequencing revealed that a total of 8133 genes were significantly altered by PVY infection, of which 3790 were up-regulated and 4343 were down-regulated (Fig. 2B, Supplementary Table S5). To confirm the RNA sequencing data, we selected 10 up-regulated genes, 2 down-regulated genes and 1 gene that was not changed via qRT-PCR to check their expression patterns compared with RNA-seq data, the qRT-PCR results were consistent with the deep sequencing data (Supplementary Fig. S2). To understand the putative roles of the significantly altered genes, GO analyses were conducted to discover their relevance to diverse biological processes, molecular functions and cellular components. Genes in PVY-infected plant were found to be enriched in metabolic and stress response processes (Fig. 4A,B,C).

### Combined analysis of miRNA and mRNA expression network under PVY infection

**Figure 5:**
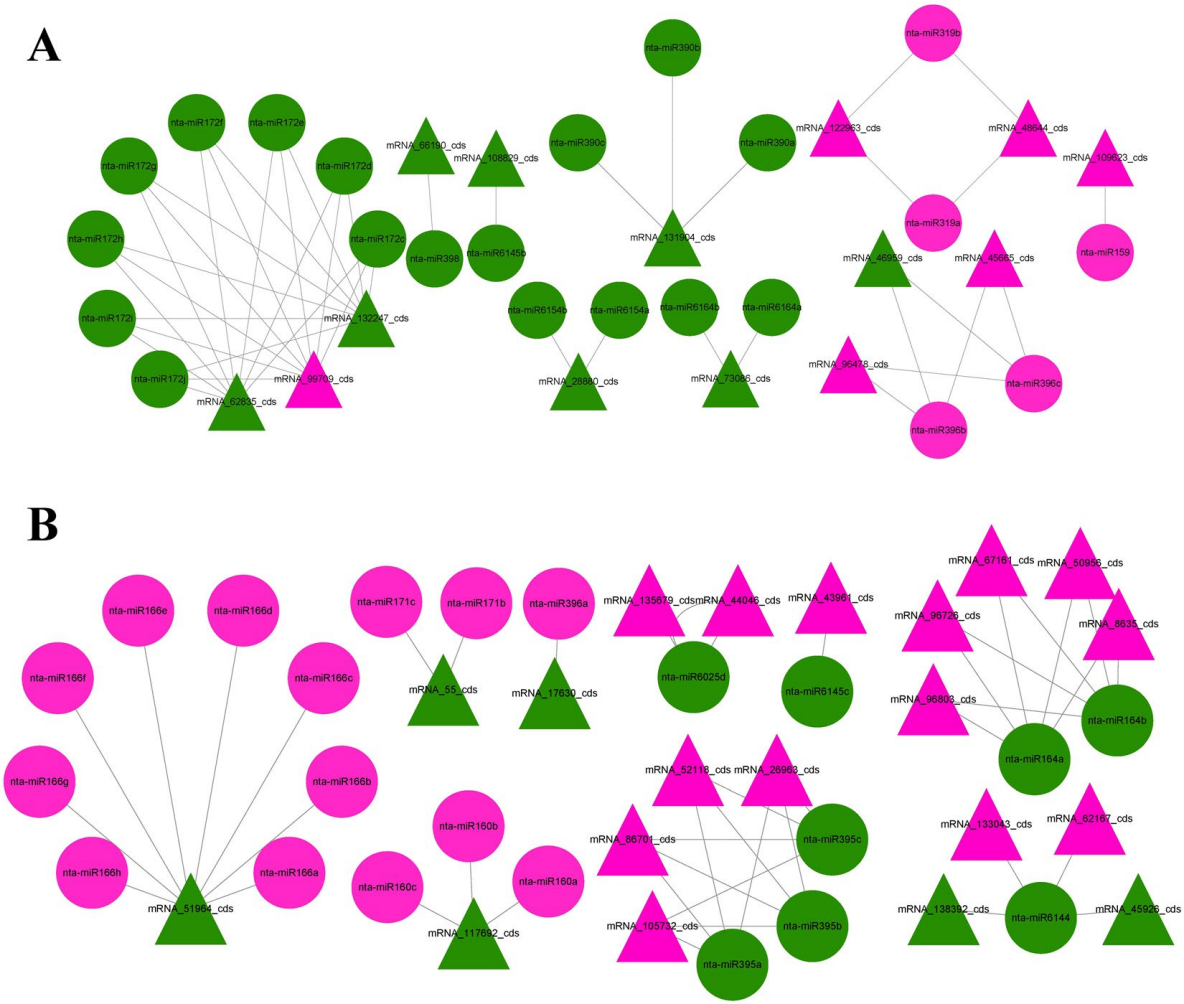
Combined analysis of miRNA and mRNA expression network under PVY infection.(**A**) Positive regulation of miRNAs versus their target genes; (**B**) Negative regulation of miRNAs versus their target genes.The triangle represents mRNA, circle represents miRNA, green and red represent down regulate and up regulate, respectively.

To explore the miRNA and mRNA expression networks in the PVY-infected tobacco plants, data on the miRNA and mRNA expression profiles were combined for a further correlation analysis. It is well known that miRNAs play pivotal roles in regulating mRNA expression. Correlation analysis of the 81 differentially-expressed miRNAs and the 8133 differentially-expressed mRNAs identified a total of 88 interaction pairs of miRNA and its corresponding targets mRNA (Supplementary Table S6). For each such pair, a one-to-one correspondence between up-regulated miRNA (or down-regulated miRNA) and down-regulated target mRNA (or up-regulated target mRNA) was not necessarily expected. So, to directly demonstrate the relationship between differentially-expressed miRNAs and mRNAs, we then classified these relationships into two categories depending on their regulation mode: either as positive or negative, respectively, for up-regulated (down-regulated) miRNAs versus up-regulated (down-regulated) target genes (Fig. 5A, Supplementary Table S7) or up-regulated (down-regulated) miRNAs versus down-regulated (up-regulated) target genes (Fig. 5B, Supplementary Table S8). The results showed that in addition to the repression caused by miRNA, other mechanisms such as ceRNA regulation might be involved. These data collectively suggested that the interactions between mRNA and miRNA are very complicated in the tobacco plant.

### PVY-derived siRNA targets the *NtTCTP* mRNA and mideate plant resistance to PVY infection

**Figure 6:**
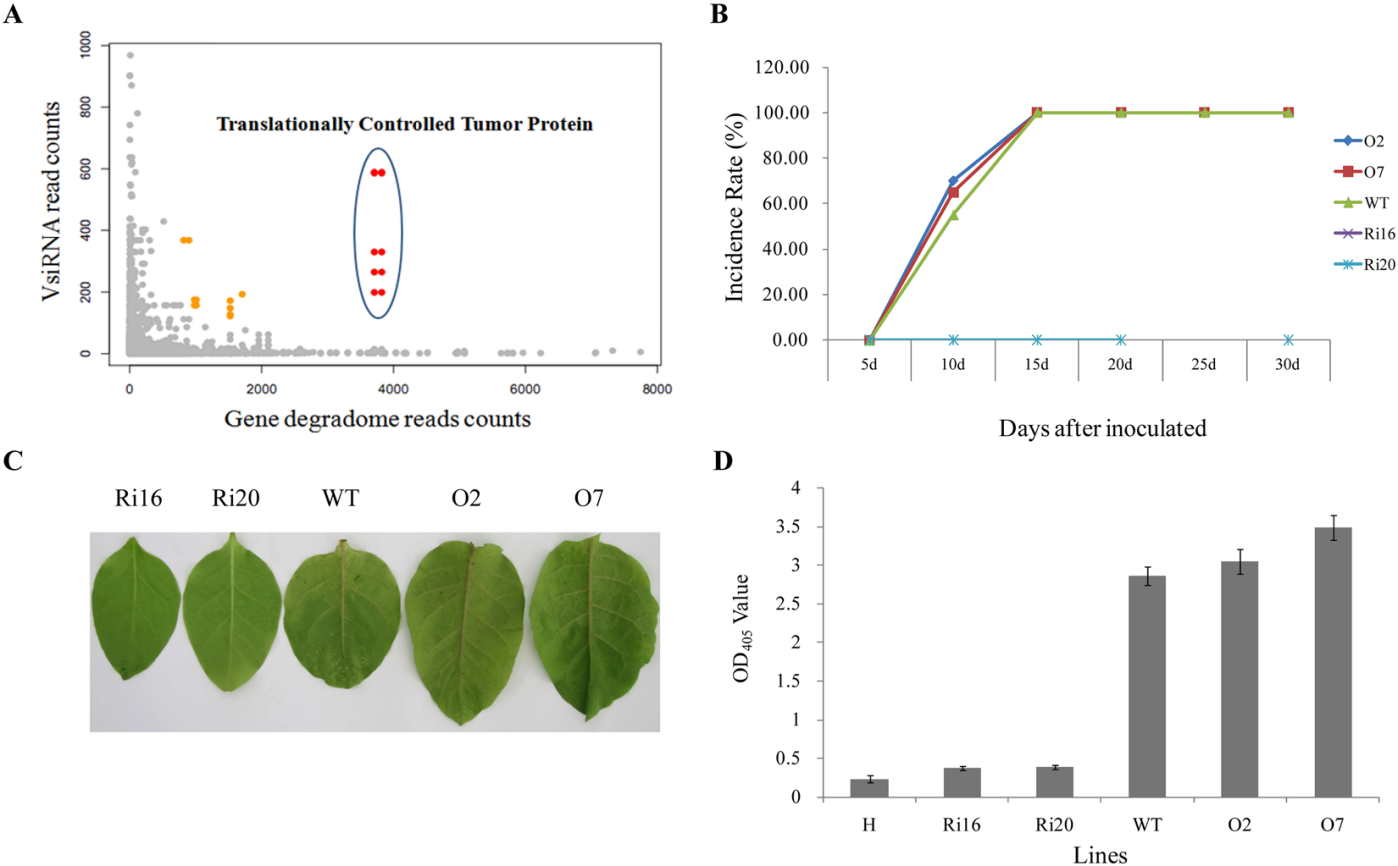
Virus-derived siRNA (vsiRNA) from PVY targets *NtTCTP* mRNA and mideates plant resistance to PVY infection.(**A**) VsiRNA from PVY targets *NtTCTP* mRNA, x-axis represents the read counts of degradome sequencing for the genes that predicted as the targets of vsiRNA from PVY genome, y-axis represents vsiRNA reads from PVY genome;(**B**) The incidence rate of *NtTCTP* overexpressing lines O2, O7,wild type and *NtTCTP* silencing lines Ri16, Ri20; (**C**) Typical symptoms of *NtTCTP* overexpressing lines O2, O7,wild type and *NtTCTP* silencing lines Ri16, Ri20.(**D**) Virus concentration of healthy plant (**H**), *NtTCTP* overexpressing lines O2, O7,wild type and *NtTCTP* silencing lines Ri16, Ri20 detected by ELISA.

To further investigate the plant response to PVY infection, we analysed the PVY-derived siRNAs and their relationships with the tobacco mRNAs. Computer-assisted analyses identified several vsiRNAs derived from the PVY genome that were complementary to the mRNA sequence of the *NtTCTP* gene (*Nicotiana tabacum* Translationally Controlled Tumor Protein, mRNA_68091) ORF (Fig. 6A). The most abundant vsRNA that targets *NtTCTP* was quantified by Northern blotting (Supplementary Fig. S3). Subsequent analysis of the degradome sequencing data also confirmed that the *NtTCTP* was excised in the PVY-infected plants, thus suggesting that vsiRNAs from PVY may down-regulate *NtTCTP* expression such that *NtTCTP* may also have an important role in plant-PVY interactions.

To test this hypothesis, we conducted a PVY infection assay with two NtTCTP-overexpressing lines (NtTCTP-OE: O2 and O7) and two NtTCTP*-*silencing lines (NtTCTP-RNAi: Ri16 and Ri20) as part of a further analysis^36^. Seedlings of O2, O7, Ri16, Ri20 and the wild type (variety *Xanthi*) were challenged with PVY at the four-leaf stage of development. After 2 weeks, PVY-induced symptoms in the systemic leaves of the wild type and in the two NtTCTP-overexpressing lines showed greater sensitivity when compared with the wild type seedlings. In contrast, the two NtTCTP-silencing lines showed high resistance to PVY infection and they did not display any obvious symptoms (Fig.6B,C). ELISA consistently detected high PVY tires in the wild type and NtTCTP-overexpressing lines, whereas PVY was hardly detected in the two NtTCTP-silencing lines (Fig. 6D). These results suggest that PVY-plant interaction involved *NtTCTP* which acted as a susceptibility factor to promote the PVY infection.

A prior study reported that NtTCTP interacts and stabilizes the ethylene receptor NtHK1 to reduce the plant response to ethylene and to promote plant growth through accelerated cell proliferation^36^. To see whether NtHK1 also participated in the tobacco plant reponse to the PVY infection, we tested several NtHK1-overexpressing (NtHK1-OE:16-4) and NtHK1*-*silencing (NtHK1-RNAi:1–8) lines. However, no apparent differences were found among the NtHK1-overexpressing, the NtHK1*-*silencing and the wild type plants after inoculation with PVY. All of those plants showed typical symptoms and their incidence rate and virus content were almost the same across the three lines (data not shown), which together suggested that NtHK1 had no function in the PVY infection of tobacco. Nonetheless, these results did demonstrate that the involvement of *NtTCTP* in the PVY-plant interaction did not occur via the ethylene pathway.

## Discussion

High throughput sequencing approaches have become powerful tools for analysing global gene expression profiles and for identifying low-abundance novel miRNAs unidentifiable by traditional cloning and sequencing techniques^37–39^. Global expression profiling analysis of miRNAs and mRNAs in the same samples can provide a unique opportunity to enhance our understanding of potential miRNA regulatory mechanisms in host-infection by virus.

RNA-seq analyses have been done for PVY-infected potato plants^40^, with these studies finding that several genes were expressed differently between the susceptible and resistant varieties. By way of comparison, our sequencing results provide a detailed view of miRNAs and mRNAs expression in tobacco leaf in response to PVY infection, thus adding new information to better understand the virus-host interactions as well as offering novel insights into the impact of viral infection on host small RNA and mRNA exprsssion.

In general, miRNA accumulation will lead to the down-regulation of corresponding mRNA targets, and vice-versa. After an integrated analysis of the differentially-expressed miRNAs and mRNAs, we found several important regulatory miRNAs likely involved in virus infection. For example, miRNA6019a was able to target a “disease resistance protein” (mRNA_90605), which is considered as one of TIR-NBS-LRR family resistance genes. In the PVY infection process, miRNA6019a was down-regulated (Supplementary Fig. S1B,C) while the amount of its target mRNA 90605 increased correspondingly (Supplementary Table S5). This coordinated activity suggests that miRNA-regulated resistance might be promoted during PVY infection.

When studying vsiRNA, how it performs key roles in antiviral resistance and in host transcripts regulation are the core questions. For example, it was reported that a siRNA derived from CMV satellite RNA could target and silence CHLI, a chlorophyll biosynthetic gene, to induce the symptoms of yellowing in virus-infected plants^27, 28^. Moreover, a vsiRNA derived from RSV (*Rice stripe virus*) targets *eIF4A* mRNA in tobacco and down-regulates its expression, resulting in a phenotype of leaf-twisting, deficient flowers and stunting^30^. In the present study, we identified several vsiRNAs derived from the PVY genome which could target the host gene *NtTCTP*. The PVY infection assay on the *NtTCTP-*silencing and over-expressing transgenic lines showed that silencing *NtTCTP* suppressed the PVY infection, whereas the over-expression of *NtTCTP* increased plant susceptibility to the PVY infection. Therefore, it is plausible *NtTCTP* encodes a host factor that is essential to the PVY infection process, not unlike for other host factors recently reviewed^41^.

TCTP is a highly conserved protein present in all eukaryotes. Its mammalian homologs are perhaps the best studied due to their role in cancer development. TCTP is an important component of the TOR (target of rapamycin) signalling pathway, the major regulator of cell growth in both animals and fungi. Though many studies have revealed that TCTP is involved in cell cycle progression, cell growth, stress protection, maintenance of genomic integrity and apoptosis^42^, its molecular function remains elusive. Recently, TCTP was suggested as an important host factor in the *Pepper yellow mosaic virus* (PepYMV) infection of tomato and *Nicotiana benthamiana* plants. This particular virus interferes with the subcellular localization of this protein, probably due to the involvement of TCTP at some crucial stage of the infection process^43^. Tobacco TCTP (*NtTCTP*) encodes a small ER-located protein containing 168 amino acids, and the transcripts of *NtTCTP* were more abundant in roots than in the other plant organs^36^. Finally, our research found that tobacco *NtTCTP* was a target of vsiRNA from the PVY genome and that the silencing of *NtTCTP* mediated resistance against PVY.

As our primal expectation, the content of PVY^N^ should accumulate increasingly in NtTCTP-silencing lines because vsiRNA derived from PVY targeted to *NtTCTP*, but the opposite results showed that *NtTCTP* was required for PVY infection. Why does the virus generate the vsiRNA which has adverse effect on itself? We speculated that targeting of *NtTCTP* by vsiRNA is a host strategy resistant against PVY infection, but the function of these vsiRNA could be suppressed to some extend by some else mechanism. It might be an episode of host-virus competition series. Further research is planned towards determining the functional role of TCTP in viral infection, and to test whether this gene can be manipulated against PVY, PepYMV and related viruses.

## Conclusion

We described the miRNA and mRNA expression profiles in virus-infected tobacco plants by using high-throughput sequencing technology. Combining these two datasets we identified an network consisting of 88 miRNA-mRNA interactions. In so doing, we further found that vsiRNA from PVY target tobacco *NtTCTP* mRNA to mediate plant resistance to PVY infection. The integrated high-throughput expression datasets we obtained provides a valuable resource to examine global genome expression changes in plant responses to PVY infection, which should also contribute to viral symptom development. This study thus offers new insights into the pathogenicity mechanisms of PVY and associated plant resistance mechanisms.

## Materials and Methods

### Plant growth conditions and virus infection

Seeds of tobacco (*Nicotiana tabacum*) were surface sterilized with a 3% sodium hypochlorite solution, rinsed five times with distilled water, immersed in distilled water for two days, and then allowed to germinate for another 2 days at 37 °C. Seedlings were grown in a medium of half-strength growth nutrients under a 16-h light (28 °C)/8-h dark (25 °C) photoperiod. The PVY^N^ (tobacco veinal-necrotic strain) was maintained in potato in green houses at 25 ± 3 °C, 60 ± 5% relative humidity under natural sunlight. Seedlings were challenged with PVY at the four-leaf stage. Two weeks later, when the virus induced symptoms appeared in the systemic leaves of the PVY-infected plants, leaves from the PVY-infected plants (three biological replicates: A1, A2, A3) as well as the mock-inoculated plants (three biological replicates: B1, B2, B3) were collected and immediately frozen in liquid nitrogen until subsequent use.

### RNA isolation and sequencing

Total RNA was extracted using the phenol-chloroform method.All samples were assessed for integrity and population size using the Agilent 2100 Bioanalyzer. The concentration and purity of each RNA sample was measured using the Nanodrop spectrophoto meter (Thermo Scientific). Small RNA and mRNA library preparation and sequencing were performed using Small RNA Sample Preparation Kit (Illumina, RS-200-0048) and NEBNext® Ultra™ RNA Library Prep Kit for Illumina® (#E7530L, NEB, USA) following the manufacturer’s recommendations.

### Analysis of miRNA and mRNA sequencing data

The filtered sequence of all the samples were mapped to tobacco genome (http://solgenomics.net/) by bowtie1^44, 45^ with –v –a -best, that is, align reads to tobacco genome without any mismatch (because of the short length of miRNA) and the all best alignments were retained for follow-up analysis. The mapped sRNA sequence would be used for the known miRNA, Rfam, repeat and some other annotation. Tobacco miRNA from miRBase 21 was used for known miRNA reference sequence. Rfam 12.0 was used for ncRNA annotation. The unannotated sRNA was used for novel miRNA prediction by miRDeep-P software.To get the differential miRNA for both known miRNA and novel miRNA, DESeq software was used. MiRNAs which satisfied FDR ≤ 0.05 and log_2_|fold change| >  = 1 were considered as significantly differential expressed miRNAs (DEM) between the two groups.Target genes of the DEMs were predicted by software psRobot.

Libraries of RNA were sequenced on the Illumina Hiseq 2500 platform and sequencing reads that contained polyA/T and adapters were discarded, and reads with low quality and high Ns were pre-filtered before mapping, too. Filtered reads were mapped to the reference Tobacco genome sequence (http://solgenomics.net/) by using Tophat with the default parameters.HTseq is applied to generate gene counts. Then gene expression difference was analyzed by DEseq, genes with |log_2_FoldChange| ≥ 1 and p-value < 0.05 were determined to be statistically significant.

Function and pathway enrichment were analyzed depending on the Gene Ontology database (http://geneontology.org/). The GO term with a q-value lower than 0.05 is determined to be enriched significantly.

### Quantitative reverse transcription real-time PCR analysis

Quantitative reverse transcription real-time PCR (qRT-PCR) was performed using a SYBR Real-time PCR Detection System (MJ Research, Waltham, MA, USA) following the manufacturer’s instructions. Each reaction was prepared in a total volume of 20 µl containing 10 µl SYBR Green Mix (Takara), 1.5 µl of diluted cDNA (corresponding to 1.5 ng of reverse-transcribed total RNA) and 0.2 µl of each primer (200 nM working concentration). The reactions were subjected to an initial denaturation step of 95 °C for 10 s, followed by 35 cycles of 95 °C for 5 s, 60 °C for 30 s and 72 °C for 10 s. Each sample was prepared in triplicate. Each sample was prepared in triplicate.The qRT-PCR primers used in this study were list in Supplementary Table S9.

### Northern blot hybridization

MiRNA northern blot hybridization was performed as described^46^ by Guo *et al*. with 30 micrograms of sRNA loaded for each sample. DNA oligonucleotides complementary to miRNA sequences were end-labeled with r-^32^P-ATP (5000 Ci mmol^−1^) using T4 polynucleotide kinase (NEB, Beijing, China) as the probe. Membranes were hybridized for 16 h at 42 °C and were briefly air-dried and were exposed to X- ray film at −80 °C.

### Target prediction of the differentially expressed miRNAs (DEMs) and degradome sequencing

To predict the target genes of the DEMs, a PsRobot was used. This tool identifies a particular set of sRNAs with stem-loop shaped precursors (such as microRNAs and short hairpin RNAs), as well as their target genes or transcripts, especially in plants. It predicts their targets using a modified Smith–Waterman algorithm. This program was performed with –ts 2 –gl 10 setting. That means when aligned with the reference genes, those DEMs at position after 10 was allowed at most one gap or bulge, target penalty score should be lower than 2. Mismatches, gaps or bulges are evaluated with a penalty of plus 1, while the G:U pairs are evaluated with a penalty of plus 0.5^47^.

The degradome library was constructed as previously described^48^ by using the RNA of the A1 treatment as the core material. Firstly, the RNA fragments with poly (A) tail were isolated from the total RNA by using the Oligo texm RNA mini kit (Qiagen), Secondly, a 5′ RNA adapter with a *Mme* I restriction site at its 3′ end was added to the 5′ ends of the isolated poly(A) RNAs. Thirdly, reverse transcription PCR using oligod (T) as the primer was performed and the PCR products were purified and digested with *Mme* I. After ligating a double-stranded DNA adapter to the 3′ end of the digested products, the ligated products were further purified and amplified, and then sequenced using the Illumina GAII platform.

## Electronic supplementary material

supporting information

Table_S1

Table_S2

Table_S3

Table_S4

Table_S5

Table_S6

Table_S7

Table_S8

Table_S9
