# Supplementary material for: Integrated analysis of tobacco miRNA and mRNA expression profiles under PVY infection provids insight into tobacco-PVY interactions
Source: Sci Rep. 2017 Jul 7;7:4895. doi: 10.1038/s41598-017-05155-w (PMC5501784; doi:10.1038/s41598-017-05155-w)
Supplement: Supplementary file 1 — supporting information [file 41598_2017_5155_MOESM1_ESM.pdf]

Title: Integrated analysis of tobacco miRNA and mRNA expression profiles under PVY infection provids insight into tobacco-PVY interactions

Authors: Yushuang Guo[1,a], Meng-ao Jia [1,a,*], Yumei Yang[2,a], Linlin Zhan[4], Xiaofei Cheng[5], Jianyu Cai[4], Jie Zhang[1],Jie Yang[2],Tao Liu[2],Qiang Fu[1],Jiehong Zhao[1] & Imran Haider Shamsi[3*]

Affiliations:

1. Key Laboratory of Molecular Genetics, China National Tobacco Corporation, Guizhou Institute of Tobacco Science, Guiyang, Guizhou, 550083, P.R.China.

2. Annoroad Gene Technology (Beijing) Co., Ltd, Beijing, 101100, P.R.China.

3. College of Agriculture and Biotechnology, Zhejiang University, Hangzhou, Zhejiang, 310058, P.R China.

4. College of Agriculture and Food Science, Zhejiang Agriculture and Forestry University, Hangzhou, Zhejiang, 311300, P.R. China.

5. School of life and Environmental Science, Hangzhou Normal University, Hangzhou, Zhejiang, 311121, P.R. China.

a: Those authors contributed equally to this work.

***Corresponding authors:** Meng-ao Jia and Imran Haider Shamsi

E-mail: jiamengao@cau.edu.cn and syedimranshamsi@yahoo.com

Supporting Information:

Table S1: Basic information of tobacco miRNAs

Table S2: Differentially expressed mRNAs in PVY-infected tobacco plants.

Table S3:Targets of differently expressed miRNAs by PVY infection

Table S4: Degradome sequencing results for the targets of miRNAs and vsiRNAs

Table S5: Differently expressed mRNAs by PVY infection

Table S6: The 88 interaction pairs of DEM and its target DEGs

Table S7:The miRNAs positively correlated with their target mRNAs

Table S8: The miRNAs negatively correlated with their target mRNAs

Table S9 : Primers used for qRT-PCR in the text.

Fig.S1. Differentially expressed miRNAs in PVY-infected and mock-inoculated tobacco plants. (A) Expression profiles from miRNA deep sequencing. Every row shows a different miRNA. Red, black and green indicate expression levels of miRNAs low,medium and high, respectively. (B) Expression profiles of miR156g, miR168a, miR 396b, miR169a, miR6149a and miR6019a. Every row shows a different miRNA. Red, black and green indicate expression levels of miRNAs low, medium and high, respectively. (C) RNA gel blots showing expression of miR156g, miR168a, miR 396b, miR169a, miR6149a and miR6019a in virus infected tobacco and mock plants.Tobacco U6 was probed as a control.

Fig.S2. Fold Changes of selective genes quantified by both deep sequencing and qRT-PCR methods.

Fig.S3. Quantifying of the most abundant vsRNA that targets *NtTCTP* in virus

infected tobacco and mock plants by Northern blotting.Tobacco U6 was probed as a control.

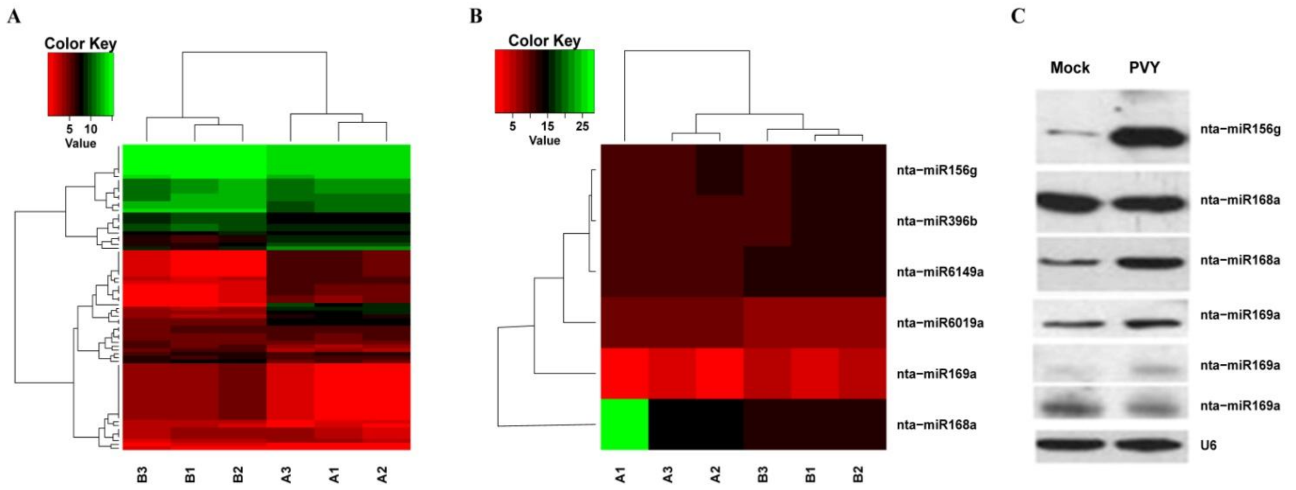

Fig. S1

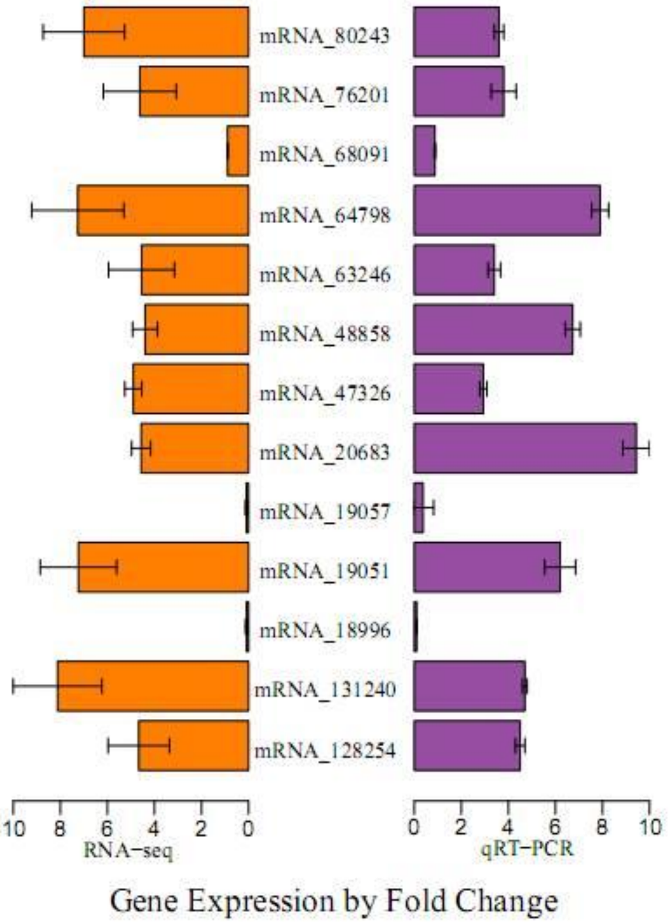

Fig. S2

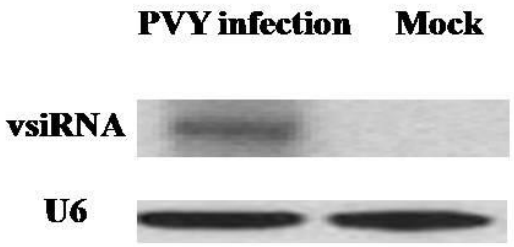

Fig. S3
